# The Nitric Oxide Donor SNAP-Induced Amino Acid Neurotransmitter Release in Cortical Neurons. Effects of Blockers of Voltage-Dependent Sodium and Calcium Channels

**DOI:** 10.1371/journal.pone.0090703

**Published:** 2014-03-05

**Authors:** José Joaquín Merino, Carmen Arce, Ahmad Naddaf, Victor Bellver-Landete, Maria Jesús Oset-Gasque, María Pilar González

**Affiliations:** 1 Departamento de Bioquímica y Biología Molecular II. Facultad de Farmacia, Universidad Complutense de Madrid (UCM), Madrid, Spain; 2 Instituto Universitario de Investigación en Neuroquímica (IUIN). Universidad Complutense de Madrid (UCM), Madrid, Spain; 3 Faculty of Pharmacy, Isra University, Amman, Jordan; Albany Medical College, United States of America

## Abstract

**Background:**

The discovery that nitric oxide (NO) functions as a signalling molecule in the nervous system has radically changed the concept of neuronal communication. NO induces the release of amino acid neurotransmitters but the underlying mechanisms remain to be elucidated.

**Findings:**

The aim of this work was to study the effect of NO on amino acid neurotransmitter release (Asp, Glu, Gly and GABA) in cortical neurons as well as the mechanism underlying the release of these neurotransmitters. Cortical neurons were stimulated with SNAP, a NO donor, and the release of different amino acid neurotransmitters was measured by HPLC. The involvement of voltage dependent Na^+^ and Ca^2+^ channels as well as cGMP in its mechanism of action was evaluated.

**Conclusions:**

Our results indicate that NO induces release of aspartate, glutamate, glycine and GABA in cortical neurons and that this release is inhibited by ODQ, an inhibitor of soluble guanylate cyclase. Thus, the NO effect on amino acid neurotransmission could be mediated by cGMP formation in cortical neurons. Our data also demonstrate that the Na^+^ and Ca^2+^ voltage- dependent calcium channels are involved in the NO effects on cortical neurons.

## Introduction

Nitric oxide (NO) is a short-lived gas produced by the family of NO synthases from the amino acid L-arginine [1]. Its identification as a signalling molecule in the nervous system changed the concept of neuronal communication [2]. NO is synthesized on demand and diffuses from nerve terminals in the vicinity of the NO producing cells. The mechanism occurs at neuronal and non-neuronal levels and NO release has pleiotrophic effects [3]–[5], suggesting that it acts as a neuromodulator and/or neurotransmitter [6], [7]. NO has also been implicated in behaviour, learning and feeding [8]–[10]. The soluble guanylate cyclase (sGC) enzyme has long been considered the major physiological target for neuronal NO [11]–[13]. Thus, nitrergic nerve stimulation or administration of NO donors increases intracellular cGMP concentrations [14]–[18] and could enhance nitrergic effects.

NO has been shown to modify the release of several neurotransmitters such as acetylcholine [19], [20], noradrenaline [21], [22], dopamine [23], glutamate [24]–[27], GABA [28]–[30], serotonin [31], adenosine [30], carbon monoxide [32] and opioids [33]. Conversely, Jin et al. [34] report that the NO precursor arginine and the NO donor SNAP reduce glutamate release from primary afferent terminals through S-nitrosylation of voltage-activated Ca^2+^ channels. On the other hand, Sistiaga et al [16] reported that NO inhibits glutamate release in cortical neurons stimulated with 4-aminopyridine. The mechanisms underlying in these effects are still not fully understood. Nonetheless, the direct S-nitrosylation of receptors, the activation of cGMP-dependent protein phosphorylation, the regulation of neuronal energy and the modulation of transporters are potential mechanisms affecting neurotransmitter release [35]–[37]. In this study, we look into the role of NO as a regulator of excitatory (Asp and Glu) and inhibitory (Gly and GABA) amino acid release in cortical neurons and the possible involvement of calcium and sodium channels on neurotransmitter release (Glu, Asp, Gly, GABA). For this purpose, we used SNAP, a NO donor to increase NO levels in cortical neurons. We evaluated the levels of different neurotransmitters in these cells (Asp, Glu, Gly, GABA) by HPLC.

## Materials and Methods

### Ethics statement

Pregnant rats were obtained from the “Laboratory Animal from the Universidad Complutense de Madrid (U.C.M)”; Licence number #ES280790000086. The work was also approved by the University Animal Care Committee (C.E.A = Commite of Experimental Research and Ethics) from the Universidad Complutense de Madrid (U.C.M; form number RD # 53/2013 for research) and it was carried out in strict accordance with Guidelines for the Care and Use of Laboratory Animals from the European Communities Council Directive (86/609/EEC). All surgeries were performed under sodium pentobarbital anesthesia, and all efforts were made to minimize suffering of animals.

### Materials

Minimum Essential Eagle's Medium (EMEM) (Bio-Whittaker), and Foetal calf serum (FCS) were purchased from Sera-Lab (Sussex, England). SNAP, ODQ, CPTIO, w-conotoxin GVIA (w-CTX GVIA), verapamil and bisoxonol (bis-[1,3-diethyl-thio-barbiturate]-trimethineoxonol), were purchased from Sigma (ST. Louis, USA) and w-agatoxin IVA (w-AGA IVA) and TTX were from Calbiochem (Darmstadt, Germany). Other chemicals were research grade products from Merck (Darmstadt, Germany).

### Methods

#### Cell isolation and culture of cortical neurons

Foetal rat brains from the Wistar rats at 19 days of gestation (E19) were used in the present study. Cortical neurons were obtained following a procedure from Segal [38] with minor modifications. Isolated neurons were suspended in EMEM containing 0.3 g/l glutamine, 3 g/l glucose, 10% foetal calf serum (FCS), 100 U/ml penicillin and 100 mg/ml streptomycin. Cells were placed at a density of 10^6^ cells/ml on plastic multiwell Petri dishes. These plates were previously treated with 10 mg/ml of poly-D-lysine, to allow the attachment of the neurons to the plates. Cortical neurons were grown in a humidified chamber with 95% air/5% CO_2_ at 37°C. After 72 hours, the culture medium was replaced by fresh medium containing 10 μM of cytosine arabinoside to prevent glial cells growth. Cell viability was tested by the trypan blue exclusion method.

Glial contamination was measured following a protocol from Figueroa et al [39] using the specific anti-GFAP antibody. Briefly, cells were incubated for 1 h with anti-GFAP antibody diluted 1∶500 in PBS at room temperature. After a further wash with PBS, anti-rabbit IgG FITC conjugated was applied and incubated for 30 min as before. The secondary antibody was diluted 1∶100 in PBS prior to addition. After a final wash with PBS, the glial cells were identified by flow cytometry analysis.

Under these conditions, the glial cells represented 9%±3% of the total cell population of the culture.

#### Measurement of amino acid secretion

The secreted amino acids (Asp, Glu, Gly and GABA) were analysed by HPLC performed following the procedure described by Márquez and coworkers [40]. Cells, after 10 culture days, were washed twice with 1 ml of Locke medium. The medium was removed and cells were stimulated for 15 min at 37°C with 0.5 ml of fresh Locke medium containing the different SNAP concentrations indicated in each case. After stimulation, we followed these steps: (i) the solution containing the released amino acids were collected (ii) the cells were lysed by adding 0.5 ml of distilled water and this suspension, containing the unreleased neurotransmitters, was centrifuged at 13000×g for 5 min. Supernatants were collected out.

The amino acid concentrations were determined by reverse-phase high-performance liquid chromatography using a precolumn derivation with dansyl chloride and UV detection at 254 nm. Peaks were integrated using a Spectraphysic integrator and then quantified and compared with standards for these neurotransmitters. The separation of dansyl derivatives was carried out using a Waters Spherisorb ODS 2 column (5 μM particle size; 15×0.46 cm).

Results were expressed as % of amino acid release as compared to the total amino acid content (amino acid in the medium plus amino acid inside the cells).

#### Assessment of cell viability (XTT tests)

This assay is based on the ability of live metabolically active cells to reduce yellow tetrazolium salt (XTT) to form an orange formazan dye. Thus, this conversion can only occur in living cells. The newly-formed formazan dye is directly quantified using a scanning multiwell spectrophotometer at a wavelength of 492 nm (reference wavelength 690). The amount of orange formazan formed, as monitored by the absorbance, directly correlates with the number of living cells. Control and treated neurons were washed with PBS and incubated with the XTT solution (final concentration 0.3 mg/ml) according to Kit manufacture's instructions. After this incubation period, orange dye solution was spectrophotometrically quantified. Results were expressed as percentages with respect to the control cells, which were considered as 100%.

#### Measurement of the membrane potential

Changes in the membrane potential were monitored with the fluorescent dye bisoxonol (bis-[1, 3-diethyl-thio-barbiturate]-trimethineoxonol), a lipophylic anion that enter cell membrane in the presence of a depolarization. The increase of bisoxonol fluorescence indicates membrane depolarization, thus allows more of the negatively charged dye to enter the cells [41]. The control and treated neurons were washed and incubated for 15 min with 0.2 μM bisoxonol in the culture medium. We used 60 mM KCl as a positive control for cell depolarization. After this exposure time, the bisoxonol was removed and the neurons washed with PBS. Fluorescence was measured at 530/25 nm excitation and 590/20 nm emission and were monitored with a FL600-BioTek spectrofluorimeter. Fluorescence intensity was expressed as arbitrary fluorescence units (AFU).

### Statistical analysis

Data are shown as mean ± SEM from either two or four independent experiments using different cultures, each experiment performed in triplicate with different cell batches (total 6–12 measurements/condition). Statistical analyses were made with a One Way ANOVA on Ranks followed by the Dunn's test. One Way ANOVA on Ranks analyses the “normality” and “variance” of the data. If both data are passed the program uses the tests for parametric data but if “normality or “variance” are failed, the program performs the Kruskal-Wallis test. A value of p<0.05 was considered statistically significant. Statistical analysis were performed using SigmaPlot 11.0 software.

## Results

### SNAP action on amino acid release

Our aim was to study whether NO (SNAP) could induce amino acid neurotransmitter release (Asp, Glu, Gly, GABA). Cortical neuron cultures were exposed to different SNAP concentrations (from 1 μM to 1 mM) for 15 minutes and the amino acid content measured by HPLC in the extracellular medium as well as inside the cells. Amino acid release was expressed as the percentage of amino acid in the external medium in comparison to the total amino acid release (outside amino acid plus that inside the cells, which was assumed to make 100%).

**Figure 1:**
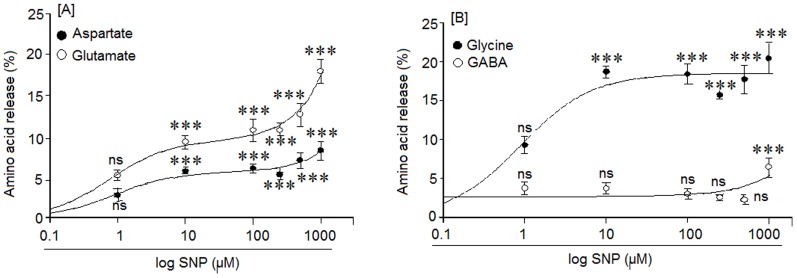
Effect of different SNAP concentrations on: [A] aspartate and glutamate, [B] glycine and GABA release, in a medium with calcium. The amino acid release was determined using an HPLC assay. Results are means ± SEM of four experiments using cells from four different cultures, each one performed in triplicate with different batches of cells (total 12 measurements/condition). Statistical significances are expressed with respect to the respective basal release, ns  =  non significant and (***)  = p<0.001. The basal neurotransmitter release levels were: Aspartate  = 3.2±0.1%, Glutamate  = 2.2±0.3%, Glycine  = 7±0.7% and GABA  = 2.3±0.4%.

The results shown in Figure 1A and 1B indicate that SNAP, at concentration between 1 μM to 1 mM, induces release of aspartate (Asp), glutamate (Glu), glycine (Gly) and GABA. The Asp release increased significantly at all concentrations tested between 10 μM to 1 mM (Figure 1A). Glutamate release remained constant at all SNAP dosages from 10 μM up to 1 mM. The maximal release of glycine was achieved at 10 μM SNAP and was always much higher than the basal values at all concentrations tested up to 1 mM. (Figure 1B). On the other hand, GABA was only released at SNAP concentrations of 1 mM (Figure 1B). The release of these amino acids neurotransmitter was Ca^2+^-dependent since SNAP had no effect in a medium without calcium (data not show).

### Action of SNAP (NO) on cell viability

**Figure 2:**
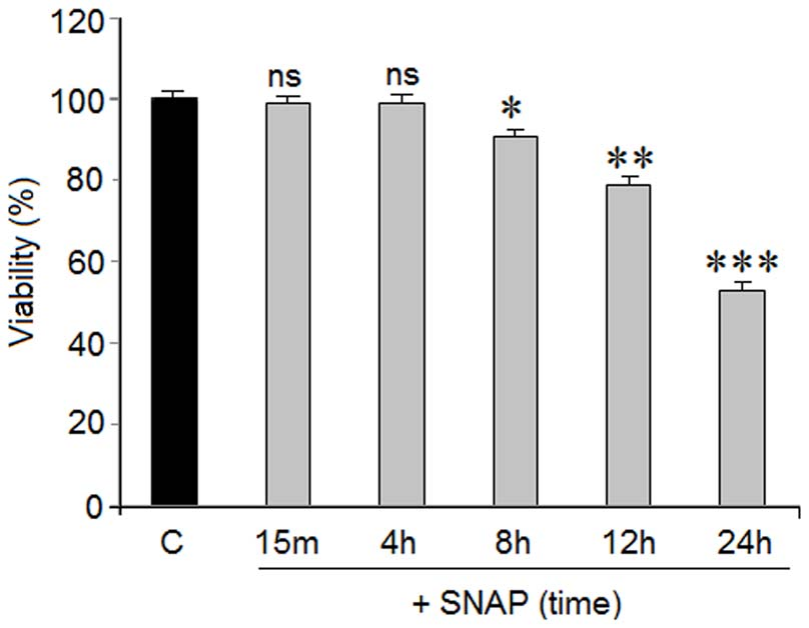
Action of 1 Cortical neurons were exposed to 1±SEM of two experiments from cells of different cultures, each one performed in triplicate with different batches of cells (total 6 measurements/condition). Statistical significances were performed with respect to the basal values. ns  =  no significant, *  = p<0.05, **  = p<0.01 and ***  = p<0.001

As it has been demonstrated that SNAP (NO) may be toxic in cortical neurons [42], we studied the action of 1 mM SNAP on cell viability at different exposure times. This experiment was performed in order to demonstrate that the amino acid neurotransmitter release mediated by SNAP was not caused by cellular death. Exposure times between 15 m to 24 hr were evaluated. Results from Fig. 2 show that cortical neurons subjected to 1 mM SNAP for 15 min to 4 hr do not present signs of lost cell viability. However, longer SNAP exposure times induced a progressive loss in cell viability that reached 40% at 24 hr.

### Effect of carboxy-PTIO on amino acid release mediated by SNAP (NO)

**Figure 3:**
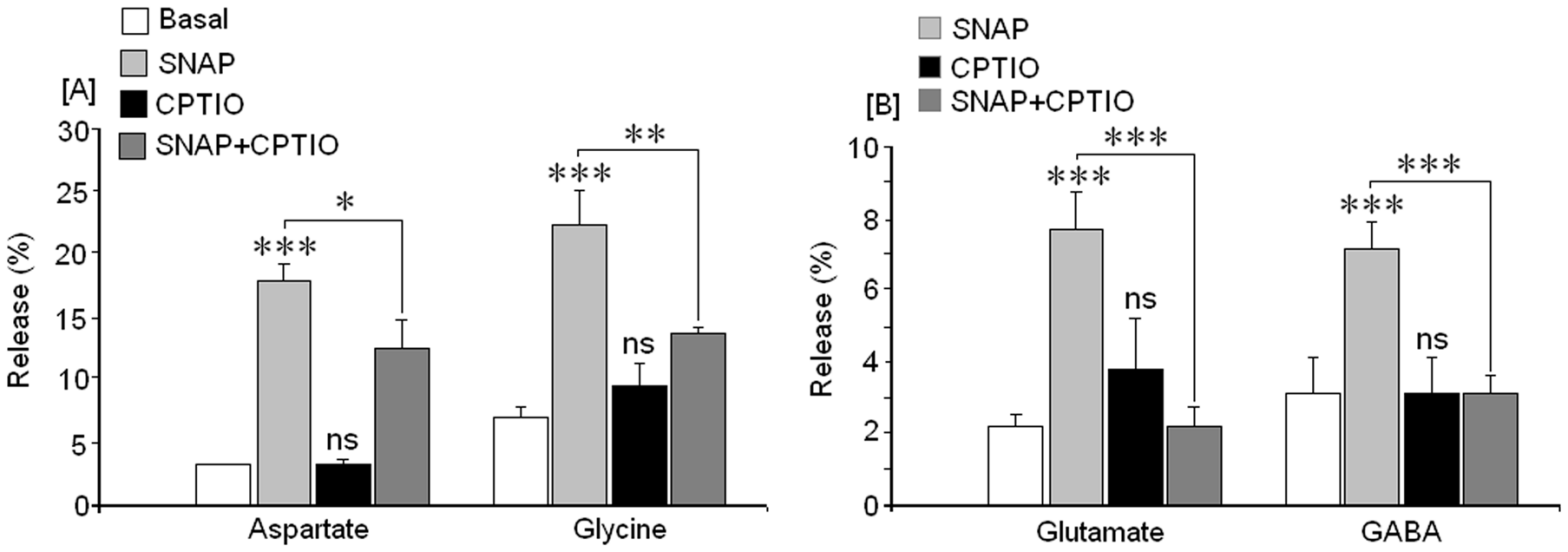
Action of carboxy-PTIO on amino acid release mediated by 1 mM SNAP. [A] Asp and Gly release, [B] Glu and GABA release. Results are means±SEM of two experiments from cells of different cultures, each one performed in triplicate with different cell batches (total 6 measurements/condition). *  = p<0.05; **  = p<0.01 and ***  = p<0.001.

As Carboxy-PTIO is a NO scavenger molecule its action on amino acid release mediated by SNAP (NO) was evaluated, in order to learn the specificity of the NO effect on SNAP-mediated amino acid release. Cortical neurones were stimulated with 1 mM SNAP in the absence and presence of 100 μM of carboxy-PTIO and the resulting amino acid neurotransmitter release was measured. Results from Figure 3A show that Asp and Gly release were inhibited in the presence of carboxy-PTIO. Similar results were found for glutamate and GABA (Fig. 3B).

### Action of guanylate cyclase on SNAP-mediated amino acid release

**Figure 4:**
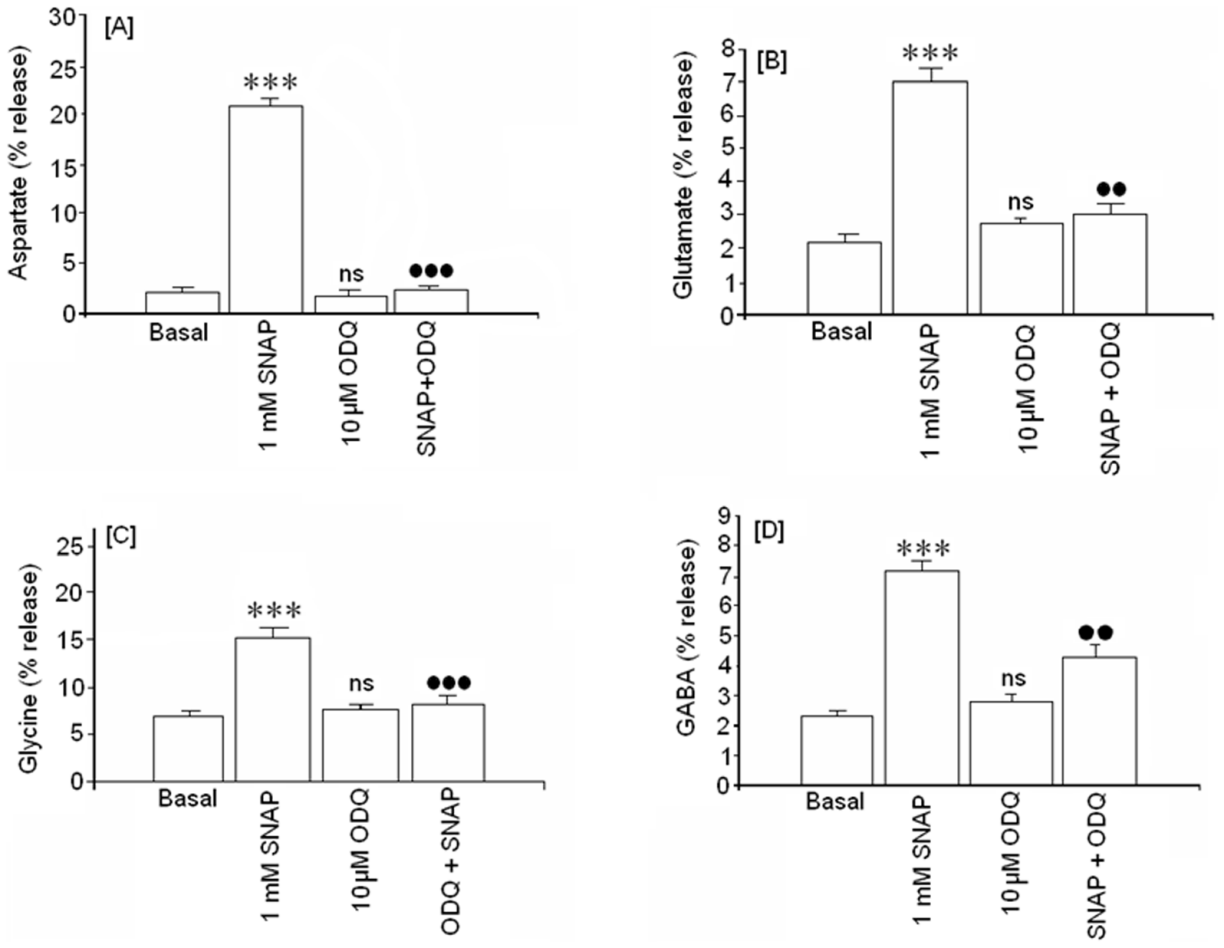
Effect of ODQ, an inhibitor of soluble guanylate cyclase, on basal and amino acid release mediated by 1 [A] aspartate, [B] glutamate, [C] glycine and [D] GABA release. Results are means ± SEM of two experiments in cells from different cultures, each one performed in triplicate with different cell batches (total 6 measurements/condition). ns or (*)  =  statistical significance as compared to basal values, ns  =  non significant. (***)  = p<0.001; (•) statistical significance between SNAP in absence and presence of ODQ. (••)  = p<0.01 and (•••)  = p<0.001.

Many NO actions have been attributed to guanylate cyclase. In order to study this mechanism, ODQ, an irreversible inhibitor of soluble guanylate cyclase (sGC), was used in the present study. We chose 1 mM SNAP concentrations to study the possible involvement of cGMP because this was the only concentration that induced GABA release in our study. Our data showed that 10 μM ODQ largely reversed the effect of 1 mM SNAP over the release of all the amino acid neurotransmitters analyzed. (Figure 4). ODQ did not significantly affect basal aspartate (Figure 4A), glutamate (Figure 4B), glycine (Figure 4C) or GABA (Figure 4D) release.

### Action of SNAP (NO) on membrane potential

**Figure 5:**
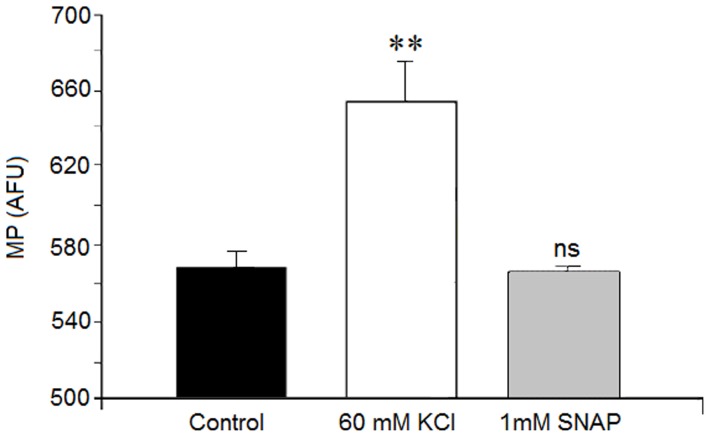
Action of SNAP on membrane potential. Neurons were treated for 15(control), or presence of either 60 mM KCl (positive control) or 1 mM SNAP. Relative changes in dye fluorescence reflect changes in membrane potential (See Material and Methods). Results are means ± SEM of two experiments in neurons from different cultures, each one performed in triplicate with different cell batches (total 6 measurements/condition). Results are shown as arbitrary fluorescent units (AFU). Statistical significance was expressed as compared to the basal value. ns  =  non significant, (**)  = p<0.01.

Cortical neurons exposure to 1 mM SNAP for 15 minutes did not alter the cell membrane potential. However, treatment with 60 mM KCl increased fluorescence (Figure 5). This fluorescence confirmed the existence of KCl-mediated membrane depolarization in the cortical neurons as well as the functionality of the assay methods.

### The possible role of voltage-dependent sodium and calcium channels on SNAP-mediated amino acid release

**Figure 6:**
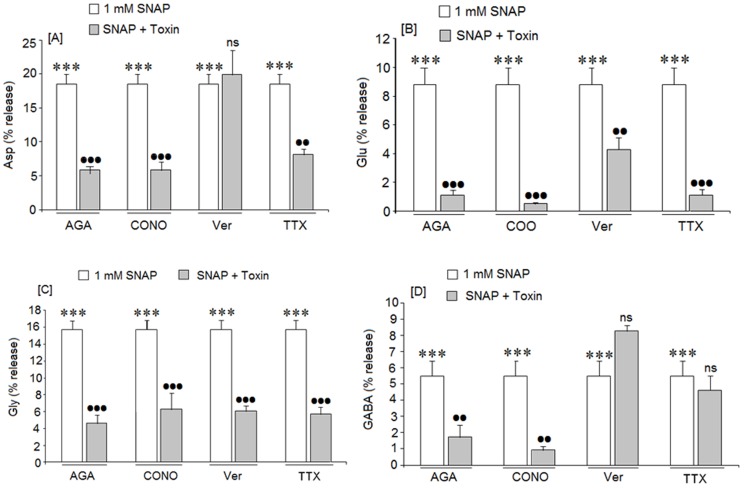
Effect of the selective inhibitors of voltage dependent Ca^2+^ and Na^+^ channels on amino acid release mediated by SNAP(NO). [A] aspartate release, [B] glutamate release, [C] Glycine release and [D] GABA release. The toxin concentrations used were w-Aga GIVA (300 nM), w-CTX GVIA (5 μM) and verapamil (10 μM); Results are means ± SEM of two experiments with cells from different cultures, each one performed in triplicate with different cell batches (total 6 measurements/condition). ns or (*)  =  statistical significance as compared to basal levels, (•)  =  statistical significance between SNAP in the absence and presence of the specific channel blocker; ns  =  non significant, (••)  = p<0.01 and (***) or (•••)  = p<0.001.

Since SNAP (NO)-mediated release of amino acid neurotransmitters is Ca^2+^ dependent, the involvement of the voltage-dependent calcium channels (VDCC) on the amino acid release mediated by 1 mM SNAP in cortical neurons was analyzed. When the action of 1 mM SNAP was assayed in the presence of w-AGA IVA, a blocker of P/Q VDCC, amino acid release was strongly inhibited (Figure 6A, 6B, 6C, 6D). Similar results were obtained with w-CTX GVIA, a blocker of N type VDCC. Nevertheless, verapamil, a blocker of L type VDCC only inhibited glutamate (Figure 6B) and glycine (Figure 6C) release without affecting aspartate (Figure 6A) or GABA release (Figure 6D).

In addition, we ascertained whether voltage-dependent Na+ channels are involved in SNAP (NO) mediated amino acid release. We used tetrodotoxin, (TTX), a toxin which blocks the voltage dependent Na+ channels (VDSC). TTX inhibited the aspartate (Figure 6A), glutamate (Figure 6B) and glycine (Figure 6C) release induced by 1 mM SNAP. However, this toxin did not affect the GABA release mediated by 1 mM SNAP in the cortical neurons (Figure 6D).

## Discussion

### NO formed from SNAP induces amino acid neurotransmitter release

We analyzed whether NO would trigger neurotransmitter release in cortical neurons. Our results showed that SNAP (NO) induces significant Asp, Glu and Gly release at SNAP concentrations ranging from 10 μM to 1 mM while GABA release was only detected at concentration of 1 mM SNAP.

This suggests that NO could regulate the balance between excitatory and inhibitory neurotransmitters in cortical neurons by through the levels of the NO donor SNAP. NO effects on excitatory and inhibitory synaptic neurotransmitter release have also been reported by several other researchers. In fact, Segieth and co-workers [43], using NO donors, found that NO regulates excitatory amino acid release in a biphasic manner in the hippocampus of freely-moving rats; Horn et al. [44], demonstrated that NO may control the activity of hypothalamic neurohypophyisis neurons because these neurons also release Glu, GABA and taurine “in vivo” after NO stimulation. On the other hand, in vivo, Engelmann et al. [45], found that NO may differentially regulate excitatory and inhibitory neurotransmitter release into the hypothalamic supraoptic nuclei. Taken together, these observations, accompanied by our findings in cortical neurons, support the hypothesis that SNAP (NO) could exert a differential effect on GABA (inhibitory) as well as glutamate (excitatory) release. The amino acid release induced by SNAP was mediated by NO because the NO scavenger carboxy-PTIO strongly inhibited the effects of the NO donor. SNAP-mediated amino acid release was not the result of cell death because exposing cortical neurons to 1 mM SNAP did not produce any loss of cellular viability (death) for at least 4 hr and our studies were performed at 15 minutes after SNAP exposure.

### Involvement of VDCCs and VDSCs in amino acid neurotransmitter release mediated by SNAP (NO)

Our results indicate that SNAP (NO) induced neurotransmitter release through calcium dependent mechanisms since there was no effect in a calcium-free medium. This means that NO probably stimulates Ca^2+^ entry necessary to provoke neurotransmitter release in cortical neurons. Calcium entry could be mediated by the action of different types of Ca^2+^ channels, such as voltage-dependent calcium channels (VDCC). In fact, when VDCC were blocked by different specific toxins, the release of all SNAP (NO) mediated neurotransmitters was inhibited in our study; since both AGA-toxin and conotoxin were effective, P/Q-and N-type VDCC probably cooperate in stimulating neurotransmitter release. The blockade of L-type VDCC by verapamil strongly reduced glutamate and glycine release without affecting aspartate or GABA release. This observation suggests that the L-type voltage dependent calcium channels do not seem to be involved in Asp and GABA release mediated by NO (SNAP), maybe because verapamil may work in a specific cell subpopulation. Differences in the involvement of different calcium channels in neurotransmitter release have been reported by others. Waterman et al. [46] indicates that parasympathetic neurons in the mouse bladder employ different Ca^2+^ channels to release neurotransmitters in these neurons. Additionally, Turner et al. [47] reported that glutamate release from whole rat brain synaptosomes was inhibited by ω-Aga toxin but unaffected by ω-CTX GIVA.

Our results on the inhibition of amino acid neurotransmitter release by the calcium channel-blocker suggests that NO induces calcium entry though VDCC since their blockade inhibits neurotransmitter release in the presence of SNAP (NO).

In addition, our results also showed that when cortical neurons were stimulated with 1 mM SNAP in the presence of TTX –a blocker of voltage dependent Na+ channels-, there was a strong inhibition of aspartate, glutamate and glycine release but GABA release was unafected. TTX data may suggest that NO activates voltage-gated Na^+^ channels and ensures VDCC and Ca^2+^- dependent neurotransmitter release. Thus, the differential sensitivity of GABA release may be related to different mechanism in GABAergic neurons were supported it by literature data [48], [49]. GABA release, resistant to Na^+^ channel blockade (TTX), has also been reported by others. Bieda and Copenhage [50], using retinal amacrine cells, found that voltage dependent Na^+^ blockade by TTX partially suppressed the Glycinergic and GABAergic input in these cells. Therefore, they concluded that amacrine cells are a cell type that uses both dependent and independent action potentials for light-evoked release of neurotransmitters. In addition, Martinez Martos et al. [51] have demonstrated that the basal GABA release in the frontal cortex of awake rats was TTX resistant.

### The activation of VDCCs and VDSC may be mediated by sGC activation

The involvement of VDCC and VDSC in SNAP (NO) release can suggest that 1) SNAP (NO) may induce depolarization and/or 2) SNAP (NO) directly modulates these channels through activation of sGC. In our study, we saw no depolarization in SNAP-treated cells. However, Mongin et al. [52] found membrane depolarization in synaptosome preparations treated with NO donors (sodium nitroprusside, S-nitroso-L-cysteine and hydroxylamine). They attributed this plasma membrane depolarization to decreased potassium permeability and inhibition of the sodium pump. Although we cannot exclude the possibility that SNAP/NO–mediated amino acid neurotransmitter release is a consequence of depolarization in cortical neurons, our results, showing the inhibitor effect of both the channel blockers and ODQ on amino acid neurotransmitter release, suggest that formation of cGMP is an intermediate step in the modulation of neurotransmitter release via GMPc on VDCC and VDNC. Although we did not directly measure VDCC and VDNC activity, the data available in the literature report that several VDCC are involved in these mechanisms [53]–[57].

**Figure 7:**
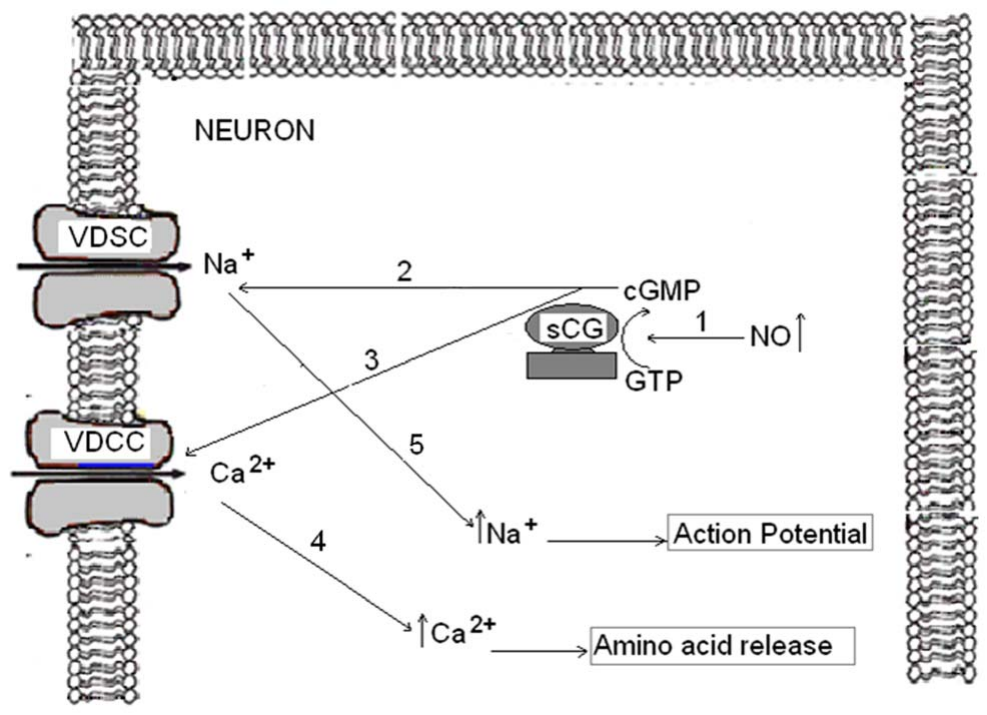
Possible mechanisms by which NO induces neurotransmitters release in cortical neurons. (1) Increased in NO induces activation of sGC and, as a consequence, cGMP formation. The increases in cGMP levels may activate VDSC (2) and VDCC (3) channels by a mechanism which yet to be elucidated. An increase in intracellular calcium levels (4) would be responsible for the amino acid release. Activation of VDSC may trigger local depolarization and promote or amplify opening of VDCC (5).

Taken together, these findings suggest that SNAP(NO) mediates amino acid release in cortical neurons (Figure 7): the NO formed from the donor (SNAP) (see 1 on Fig. 7) binds to its intracellular receptor, the soluble guanylate cyclase (sGC) activating it. The activation of sGC forms cGMP, which may directly activate Na^+^ (see 2 in Fig.7) and Ca^2+^ voltage dependent channels (see 3 on Fig. 7) by a mechanism which needs to be further elucidate. This effect would promote activation of these channels and the entry of Ca^2+^ (see 4 on Fig.7) and Na^+^ (see 5 on Fig. 7) to induce neurotransmitter release. Finally, the increase of Ca^2+^ through VDCC may be involved in this neurotransmitter release (see 4 on Fig. 7) while the increase of Na+ via VDSC would have induced action potentials in cortical neurons ¨in vitrö (see 5 on Fig. 7).

The working model presented in Figure 7 is supported by several observations from our study: 1) SNAP (NO) induces amino acid release that is inhibited by the presence of ODQ (an inhibitor of sGC) and 2) voltage dependent calcium channels of the types N, P/Q and L and voltage dependent Na+ channels are involved in the amino acid release induced by SNAP (NO).
